# Overdiagnosis: An Important Issue That Demands Rigour and Precision: Comment on "Medicalisation and Overdiagnosis: What Society Does to Medicine"

**DOI:** 10.15171/ijhpm.2017.24

**Published:** 2017-02-25

**Authors:** Stacy M. Carter

**Affiliations:** Centre for Values, Ethics and the Law in Medicine, Sydney School of Public Health, The University of Sydney, Sydney, NSW, Australia.

**Keywords:** Overdiagnosis, Medicalisation, Overtreatment

## Abstract

Van Dijk and colleagues present three cases to illustrate and discuss the relationship between medicalisation and overdiagnosis. In this commentary, I consider each of the case studies in turn, and in doing so emphasise two main points. The first is that it is not possible to assess whether overdiagnosis is occurring based solely on incidence rates: it is necessary also to have data about the benefits and harms that are produced by diagnosis. The second is that much is at stake in discussions of overdiagnosis in particular, and that it is critical that work in this area is conceptually rigorous, well-reasoned, and empirically sound. van Dijk and colleagues remind us that overdiagnosis and medicalisation are not just matters for individual patients and their clinicians: they also concern health systems, and society and citizens more broadly.

In their 2016 article, van Dijk and colleagues^1^ set out to consider the relationship between medicalisation and overdiagnosis.^2,3^ As the authors note, medicalisation is generally understood to be an older concept with origins in the social sciences,^2,4,5^ and overdiagnosis a newer concept arising from within the health and medical professions and medical sciences.^6-8^ At present, philosophers of medicine, ethicists and health researchers are actively developing definitions of overdiagnosis and attempting to distinguish overdiagnosis from similar concepts such as overtreatment, overdetection, ‘too much medicine’ and medicalisation.^2,9-16^ Throughout this commentary I will rely on Hofmann’s conception of medicalisation, as: *how phenomena, authority, or rationality related to medicine become pervasive to areas previously not considered to belong to the realm of medicine.*^3^

Conceptualising overdiagnosis is no small task. An overdiagnosis is, loosely, a correct diagnosis that, on balance, causes harm (or at least fails to benefit). However, this loose definition requires explication, and many conceptual challenges remain unresolved. At present scholars are debating whether, for example, overdiagnosis should be defined at an individual or a population level,^2,10,13,14^ whether overdiagnosis should be limited only to diagnosis of harmless disease,^9,10,13^ how benefits and harms should be conceptualised and measured and who should determine which benefits and harms ‘count,’^10,12,14^ what types of overdiagnosis might exist,^9,15^ and how to distinguish correct from incorrect diagnosis.^10,12-14^ (And these debates connect back to older debates in the philosophy of medicine about the definition and conceptualisation of disease,^17^ and in sociology about diagnosis.^18^) While acknowledging current debates in the field, I will employ the definition of overdiagnosis that I have developed with colleagues^14^:

*Consider a condition prevalent in a population, customarily labelled with diagnosis A. We propose that overdiagnosis is occurring in respect of that condition in that population when:*

1. *
the condition is being identified and labelled with diagnosis A in that population (consequent interventions may also be offered);
*
2. *
this identification and labelling would be accepted as correct in a relevant professional community; and
*
3. *
the resulting label and/or intervention carries an unfavourable balance between benefits and harms.
*

*…Benefits and harms occur at the level of individuals and populations; citizens, patients and experts have a role in identifying and weighting relevant benefits and harms.*

This definitional work is not for its own sake: it has consequences. Scholars, researchers, clinicians, and citizens care about overdiagnosis (when they do) because it is taken to cause harm and/or waste precious healthcare resources. In the paradigm case of breast cancer screening, for example, a conservative estimate suggests that for every one woman whose life is saved by mammographic screening, another three are diagnosed with a breast cancer, and receive the arduous treatment that follows, unnecessarily.^19^ Those who work within breast screening often reject the clinical and moral significance of this claim, in part because of disagreements over what counts as correct diagnosis, and what benefits and harms should matter.^20^ Much is at stake; it thus seems to me that overdiagnosis in particular needs to be approached with care and precision.

Which brings me to the article at hand. Many of the fundamental points that authors assert or rely on I endorse and would consider somewhat axiomatic: for example, that medicalisation and overdiagnosis are both social processes with social and cultural causes, that, more fundamentally, disease and its diagnosis are to some extent socially constructed, and that medicalisation and overdiagnosis occur as a matter of degree rather than dichotomously. They are right to note that sometimes in the overdiagnosis literature a strictly realist ontology of disease is implied, although increasingly the problems with this way of thinking are being pointed out.^3,13,14^

I want to focus on my concerns about the handling of the three ‘case studies,’ concerns that go to my earlier point about what is at stake, and thus, the importance of precision in conceptualisation and argumentation. The three cases are: (1) care for persons with intellectual disability; (2) diagnosis of Alzheimer disease and mild cognitive impairment; and (3) medicalisation of childbirth, particularly rising rates of caesarean section. (I note that the language used by the authors in discussing persons with intellectual disabilities would be rejected by most disability activists, and has been officially abandoned within terminological standards including the DSM-5^21^ and ICD-11.^22^ This may be a problem in translation.) The authors make a number of observations regarding these cases, and assertions based on their observations. I was not always convinced of the strength of the empirical support for the observations offered, or of a strong link between the purported observation and the corresponding assertions; my concerns, I believe, illustrate some important general issues in thinking about overdiagnosis and medicalisation.

With respect to intellectual disability in the Netherlands, the authors first claim that rates of diagnosis have not increased over the last decade, thus overdiagnosis is not occurring. This conclusion does not follow. Recall that overdiagnosis is correct diagnosis which delivers an unfavourable balance of benefits to harms; assume that intellectual disability is being diagnosed correctly according to the agreed standard. To determine whether this is overdiagnosis, we need to know what benefits and harms are produced. The standard for correct diagnosis may be set at a point where not enough people are diagnosed, and so some miss out on much-needed services and treatments. If so, incidence would be stable, but underdiagnosis would be occuring. Conversely, the diagnostic standard may be set such that many people, on balance, would have been better off if left undiagnosed (that is, on balance they are harmed more than benefited). If so, incidence would be stable, but overdiagnosis would be occurring. This cannot be determined without outcomes data. (This same problem arises in the case of Altzheimer disease discussed below and I will not note it again there.)

The authors go on to suggest that care for people with intellectual disability is becoming more expensive because more people, with less severe levels of disability, are spending time in inpatient care (the latter seems out of keeping with OECD trends, although the definition of ‘institution’ and ‘inpatient’ is not clear from the text). Again there is a question about outcomes. It is conceivable that both more money spent, and more inpatient days, could produce better health and wellbeing for persons with intellectual disability if, for example, it meant they were finally receiving vital health services. All relevant benefits and harms would need to be assessed to determine whether overdiagnosis and/or overtreatment are occurring. The authors speculate that social forces may be driving an increase in inpatient care for persons with intellectual disabilities: this may be so, but it is an empirical question that can only be answered with good quality social research.

With respect to Alzheimer disease and mild cognitive impairment, the authors make some straightforward points, especially that the disease and its thresholds are socially constructed, and that there is no clear boundary between impairment and normal ageing. The authors note that in different countries and regions there are different rates of: (1) institutionalisation of persons with Alzheimer disease; and (2) prescription of drugs to treat this disease. They conclude that it is not possible to determine which of these interventions is more medicalising. This illustrates the importance of carefully defining concepts. A rigorous conceptualisation of medicalisation should be able to do the work of identifying medicalisation (or the degree of medicalisation occurring). Employing Hofmann’s conception, for example, would prompt three questions: (1) Was cognitive impairment in older age previously considered to be in the ‘realm of medicine’?; (2) Have ‘phenomena, authority, or rationality related to medicine become pervasive’ in relation to cognitive impairment in older age? (3) If so, to what extent is this true for (*a*) institutionalisation and (*b*) pharmaceutical treatment?^3^ This should permit a determination of whether medicalisation is occurring as a result of each intervention. Note, however, consistent with the authors’ earlier arguments, that medicalisation in this context may or may not be a bad thing.^2,3,5^

The final case study is that of childbirth, particularly the use of caesarean section. The authors propose that the medicalisation of childbirth has involved many actors, and is a continuum rather than a dichotomy, both reasonable claims about medicalisation in general.^4^ They note the World Health Organization (WHO) recommendation that “no reduction in maternal and newborn mortality outcomes at the population level are found at a [caesarean section rate] higher than 15%.” This exemplifies the type of reasoning from outcomes I have been advocating throughout this commentary. The authors conclude, however, that higher rates of caesarean section suggest overdiagnosis. This would seem probable if caesarean section was a diagnosis; more precisely, it seems possible evidence of overtreatment (although again the question of which outcomes matter, and to whom, becomes important).

Perhaps the authors’ most important contribution is to gesture towards overdiagnosis and medicalisation being causally connected, and having both macro and micro manifestations. I suspect that the meso level is at least as relevant as the macro level in the social dynamics of overdiagnosis in particular (for example, committees forming guidelines, professional medical associations, local and regional health systems). Nonetheless, it is increasingly observed that overdiagnosis and medicalisation cannot be understood or addressed only at the level of the individual citizen and their clinician.^2,13,14^ This has implications for normative analysis of these problems. In particular, to quote Morrison: “*while … overdiagnosis affects individuals … it is a problem that operates at the level of systems of healthcare and has implications for social justice*.”^2^

Overdiagnosis seems likely to be a pressing challenge for some time.^14,16^ In contrast, it has been proposed that medicalisation is losing, or perhaps has already lost, its analytic force.^3,5^ As scholars studying these social processes, it is critical that we employ rigorous conceptualisation and reasoning, and sound empirical evidence, to ensure that our work moves the debate forwards and, ultimately, delivers benefit to citizens.

## Ethical issues

Not applicable.

## Competing interests

Author declares that she has no competing interests.

## Author’s contribution

SMC is the single author of the paper.
